# Animal versus human oral drug bioavailability: Do they correlate?

**DOI:** 10.1016/j.ejps.2013.08.018

**Published:** 2014-06-16

**Authors:** Helen Musther, Andrés Olivares-Morales, Oliver J.D. Hatley, Bo Liu, Amin Rostami Hodjegan

**Affiliations:** a Simcyp Limited (a Certara Company), Blades Enterprise Centre, John Street, Sheffield S2 4SU, UK; b Centre for Applied Pharmacokinetic Research, School of Pharmacy and Pharmaceutical Sciences, University of Manchester, Manchester, UK

**Keywords:** First in man pharmacokinetics, Oral drug absorption, Drug development

## Abstract

Oral bioavailability is a key consideration in development of drug products, and the use of preclinical species in predicting bioavailability in human has long been debated. In order to clarify whether any correlation between human and animal bioavailability exist, an extensive analysis of the published literature data was conducted. Due to the complex nature of bioavailability calculations inclusion criteria were applied to ensure integrity of the data. A database of 184 compounds was assembled. Linear regression for the reported compounds indicated no strong or predictive correlations to human data for all species, individually and combined.

The lack of correlation in this extended dataset highlights that animal bioavailability is not quantitatively predictive of bioavailability in human. Although qualitative (high/low bioavailability) indications might be possible, models taking into account species-specific factors that may affect bioavailability are recommended for developing quantitative prediction.

## Graphical abstract

**Figure:**
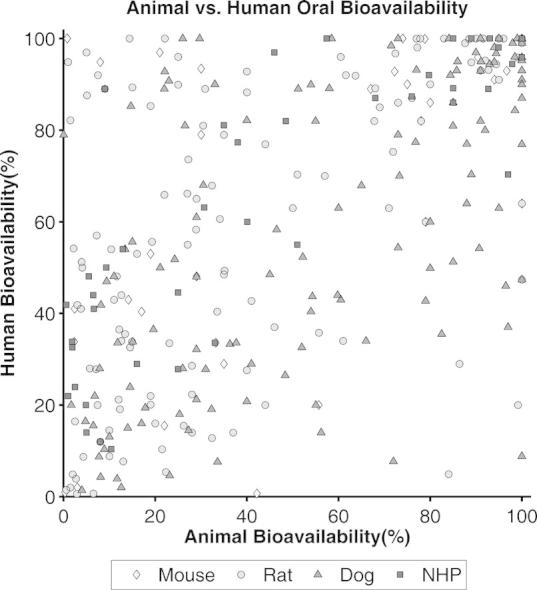

## 1 Introduction

The understanding of the absorption of oral dosage forms is a key consideration in drug development. Oral routes are preferred for being less invasive and more physiological and due to ease of administration and patient compliance. However, compared to the direct entry of the drug to systemic circulation that is achieved through intravenous dosing, additional elements affecting the availability of the drug following oral administration must be considered. These may include potential for degradation in stomach or gut lumen, metabolism in the gut wall and liver, permeability through the gut wall and incomplete release of the drug from the formulation. The molecular structure of the drug and constituents of the dosage form can determine many of these processes and they define how much of a drug reaches the systemic circulation. With all of these factors in mind, the OrBiTo project is aiming to deliver rational methods and a framework for predicting how orally-administered drugs will perform (OrBiTo, 2012). In doing so, it is important to recognise some of the current practices related to estimation of the oral drug bioavailability in humans and their validity.

Understanding oral bioavailability is not just a drug development issue but it has regulatory implications as defined by the many agencies such as FDA in their guidance for industry (FDA, 2003). These usually distinguish between the rate and extent which the active ingredient or active moiety is absorbed from a drug product and becomes available at the site of action. Since measurement at the site of action is not practical, bioavailability calculation for extravascular administration acts as a surrogate to determine the amount of drug reaching site of action relative to those from intravascular administration (Sietsema, 1989).

Subtle differences in the methods of calculating bioavailability exist which may give rise to variable results for a given drug or drug formulation. Without an understanding of these assumptions, comparison of various bioavailability measures would not be prudent. In the current drug development paradigm, administration of drugs in various preclinical species prior to human clinical studies is common for variety of reasons. It is often assumed that data on drug absorption from animals could provide reasonable estimates of bioavailability in humans. However, whilst similarity of permeability and fraction absorbed to gut wall between animals and human is established (Chiou and Barve, 1998; Chiou et al., 2000; Chiou and Buehler, 2002; Cao et al., 2006) there are considerable interspecies differences in first-pass gut and liver metabolism. These differences can prevent concluding a level of overall bioavailability in humans based on the animal data. Comparisons and correlations of human and animal bioavailability have previously been reported in the literature, and although these seem to indicate that animal values are not predictive of human bioavailability, they have mainly been limited to small sets of measurements, or comparisons within one species. In some instances queries have been raised regarding the treatment, analysis and sources of the data forming the reports. Furthermore, it is not clear whether formulations have been matched when comparing human with animal bioavailability; e.g., oral doses may have been given via solution or suspension to animals while human studies make use of solid formulations which might result in formulation-linked bioavailability differences rather than solely a species difference. There may also be differences in study design such as use of the same or different study groups for oral and iv administration which may cloud the comparisons. These issues should not be overlooked when making comparisons.

We report an extensive analysis of the published data conducted as part of OrBiTo project to clarify the relationships between human and animal bioavailability, paying specific attention to those issues described above. It is expected that this report contributes to providing an answer to the question that whether a correlation exists between the bioavailability in animals and humans and whether such animal data can be used for predicting human bioavailability; quantitatively or qualitatively.

## 2 Methods

### 2.1 Calculation of bioavailability

The overall bioavailability is often considered as a composite function of fraction released and absorbed into gut wall (*F*_abs_), fraction escaping first-pass gut wall metabolism (*F*_G_) and fraction escaping first-pass hepatic metabolism (*F*_H_):

(1) $$F=F_{\text{abs}}\times F_{\text{G}}\times F_{\text{H}}$$

Calculation of oral bioavailability (*F*), and the definition of the fraction absorbed *F*_abs_ (which is one of its three components), is not unified. Pang and Rostami have recently commented on these (2011). Whilst one may consider the total oral drug bioavailability based on deducting the fraction “unabsorbed” (1 − *F*) via analysis of feces, in many cases the dose normalised relative area under the curve (AUC) after oral and iv administration is used as a measure of oral bioavailability.

There are implications in certain situations for using each of the above methods however in general they should produce the same results. Disparities might occur when there are significant elements of entero-hepatic recirculation or high first-pass metabolism in lung. When the *F*_abs_ is defined as the fraction of given dose that passes through the gut wall, the integration of all the mass transfer (alongside the GI tract) over the time period that absorption is happening may include the drug that originates from entero-hepatic circulation. This leads to an apparent *F*_abs_ can become higher than 1 when traditional comparison of AUC after iv and oral administration is used to assess bioavailability (hence *F* could be greater than 1).

Considering the differences between definitions used to determine *F*, it was essential to pay attention to methodologies used for calculating oral bioavailability before making comparisons between various species.

### 2.2 Sources for human and animal bioavailability values

A number of reports have previously compared human and animal bioavailability values for series of compounds. One of the commonly known comprehensive reports carried out by Grass and Sinko (2002), utilising the dataset published by Sietsema (1989). There has been no attempt to expand the data within the 2002 report with any additional data published since then or refine some ambiguities in the original report. Anecdotal evidence indicated that the number of data points in the published comparisons (within a scatter graph) were not consistent with the number of compounds that appeared in the original dataset. The reasons for this were not immediately clear from the description given in the report. To assess the number of data points and their consistency with original source, the scatter plot of human vs. animal bioavailability in Grass and Sinko (2002) was digitised using GetData *Graph Digitizer v2.22* (Get Data Graph Digitizer, 2012), and the extracted data compared to that published in the original study by Sietsema (1989). In addition, the relationships between human and animal bioavailability, reported in this original database (Sietsema, 1989) were reviewed. References sources were obtained where available and checked against criteria developed for “inclusion” which ensured the values and the species were relevant to current study. Some studies were marked as ‘Rodent’ which were considered too broad in light of currently utilised preclinical species. Hence, all data relating to species other than mouse, rat, dog and non-human primates were discarded.

Additional compounds were identified using the human bioavailability database published by Varma et al. (2010). Some information on were obtained from other human vs. animal literature reports (Chiou and Buehler, 2002; Cao et al., 2006; Akabane et al., 2010). Where original data and references were not provided in the publication, the authors were contacted and invited to clarify the sources of information.

Finally, systematic literature searches being carried out using PubMed and Google Scholar for the bioavailability values in human and their corresponding animal data. Original references were obtained and inspected in all cases.

### 2.3 Inclusion and exclusion criteria

Inclusion criteria (Table 1) were applied to ensure integrity of the data and consistency between various researchers conducting the reviews. Mean bioavailability values were extracted directly from the publications. If iv and oral data had not been obtained from the same individuals and they were from different studies, bioavailability measures were considered unreliable due to potential effects of inter-subject variability. Where more than one dose was reported, the bioavailability for the lowest dose was selected in order to minimise the potential impact of saturation effects. Information on formulations were recorded. The details of strain and sex of animals utilised were noted for each reference, along with parameters relating to the compound type and use. Additional information were noted if considered beneficial to the aims and objectives of the current investigation (e.g. number of subjects where more than one reference was found) and recorded in a ‘comments section’ of database. Studies relating to controlled release formulations were discarded.

### 2.4 Statistical analysis

For compounds with more than one bioavailability study, the weighted mean for the oral bioavailability was estimated by

(2) $$\overset{¯}{x}=\frac{\sum_{i=1}^{n}w_{i}x_{i}}{\sum_{i=1}^{n}w_{i}}$$

where *x_i_* was the mean oral bioavailability for the *i*th study, *n* in the number of studies, and the weights (*w_i_*) were the number of subjects in the *i*th study, respectively. For studies with an unknown number of subjects, the assigned weights corresponded to the median number of subjects employed in the rest of the studies, with values of 6 and 5 for the human and animal studies, respectively.

Linear regression was performed for oral bioavailability in animal species (*F_Animal,species_*) and human (*F_Human_*) and the coefficient of determination (*R*^2^) and the linear regression equation were recorded for each species and the whole dataset. A similar analysis was performed by grouping the compounds by ion class (*F_Animal,ionclass_* and *F_Human,ionclass_*), and formulations (*F_Animal,formulation_* and *F_Human,formulation_*). In addition, accuracy of the *F_Human_* prediction from *F_Animal,species_* data was assessed by the ratio between animal and human oral bioavailability (*R_A/H_*) and average fold error (*afe*), Eqs. (3) and (4), respectively, whereas, for evaluation of the precision of the prediction, the concordance correlation coefficient (ccc) was calculated (Graham et al., 2012). All the statistical calculations where performed with the Statistical Toolbox within Matlab R2012a (The MathWorks Inc., Natick, MA, USA) and Microsoft Excel 2010 (Microsoft Corporation, Redmond, WA, USA).

(3) $$R_{\frac{A}{H}\text{,}i}=\frac{F_{\mathrm{Animal}\text{,}\mathrm{species}\text{,}i}}{F_{\mathrm{Human}\text{,}i}}$$

(4) $$\mathrm{Afe}=10^{\left(\frac{1}{n}\Sigma \log(R_{\frac{A}{H}\text{,}i})\right)}$$

## 3 Results

### 3.1 Data extracted from previous reports of human vs. animal bioavailability

Digitisation of the scatter plot of human vs. animal bioavailability in the report by Grass and Sinko (2002) and comparisons to the information within the tables provided by Sietsema (1989) confirmed that there were more data points than compounds in the graph for all species (Table 2). It is worth noting that although Sietsema collated bioavailability data from the literature for over 400 drugs, the human versus animal correlations were limited to approximately 70 compounds in total where the experimental bioavailability values were available for human and at least one of the other species.

Visual inspection of the tables, extracted data and the plot suggests that multiple points may have been plotted for each compound where a large range for bioavailability was reported, further detail on the analysis of these data were not available for inspection.

### 3.2 Literature search and description of the dataset

**Fig. 1:**
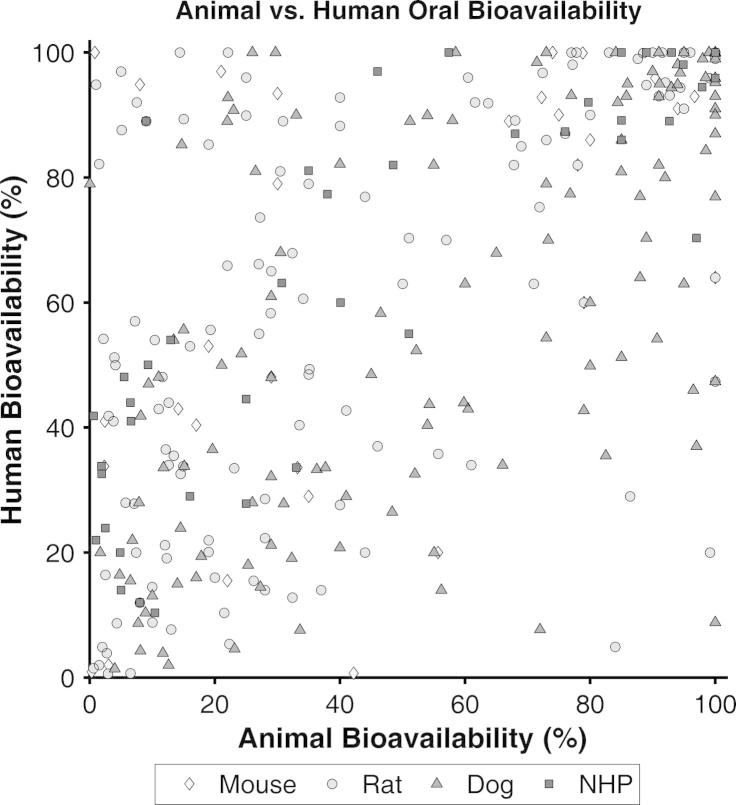
Plot of oral bioavailability (*F*) in animal species vs. oral bioavailability in humans (in percentage). Diamonds are for mouse, circles for rat, and triangles for dog and squares for non-human primates (NHP).

The literature search resulted in a total of over 1000 studies, published between 1969 and 2012, representing around 450 different compounds. The compounds with no *F_Human_* and/or the corresponding *F_Animal,species_* data were removed from the dataset. From the original dataset, only 184 different compounds with both human and animal oral bioavailability were identified and 54 of those compounds had more than one study for animal and/or human oral bioavailability. For the latter compounds, the weighted mean was calculated as described above. Finally, the number of paired datasets with both animal and human bioavailability by species was 30, 122, 125 and 41 for the mouse, rat, dog and non-human primates (NHP), respectively. In addition*, F_Animal,species_* was plotted against *F_Human_* (Fig. 1) and the final dataset is shown in Table 3. Within species, the most frequent strains employed for the oral bioavailability studies in animals were Sprague-Dawley and Wistar for the rat (49% and 35%, respectively), Beagle and Mongrel for the dog (66% and 19% respectively) and Rhesus and Cynomolgus monkey for the NHP (42% and 40%, respectively), whereas for the mouse, no clear tendency was shown for the use of any particular strain. In relation to ionic class, the majority of the compounds in the dataset were basic followed by neutral, acidic and zwitterionic, representing 50.0%, 24.5%, 15.8%, and 9.8% of the total compounds, respectively. The predominant formulation was solid (tablet and capsule) for human studies and liquid (solution and suspensions) in the case of animal studies; however for a large number of studies the formulations employed were not informed.

### 3.3 Correlation between animal and human bioavailability

**Fig. 2:**
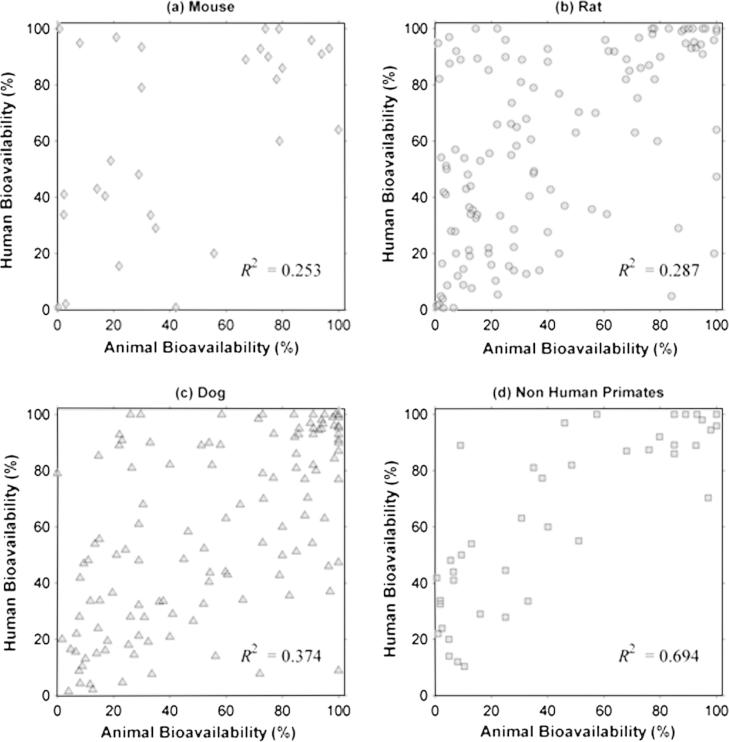
Plots for the linear regression analysis by separated by species (in percentages), the coefficient of determination (*R*^2^) for the linear regression are shown in each plot. (a) Mouse *F* vs. human *F*_;_ (b) Rat *F* vs. human *F*_;_ (c) Dog *F* vs. human *F* and (d) Non-human primates (NHP) *F* vs. human *F*.

**Fig. 3:**
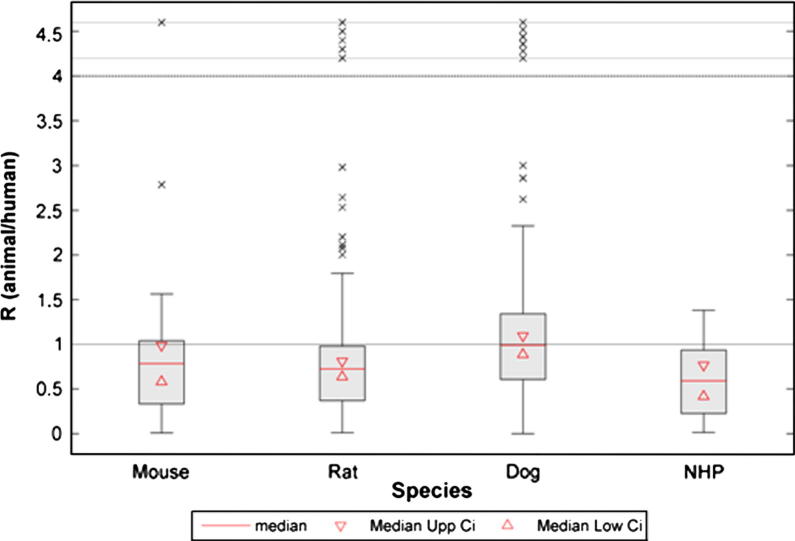
Box plots of median animal/human bioavailability ratios (*R*_A/H_) and interval between animal and human oral bioavailability. Triangles indicate 95% confidence interval (CI) for the median values; Dashed line (- - -), indicate the upper limit for outliers representation.

**Fig. 4:**
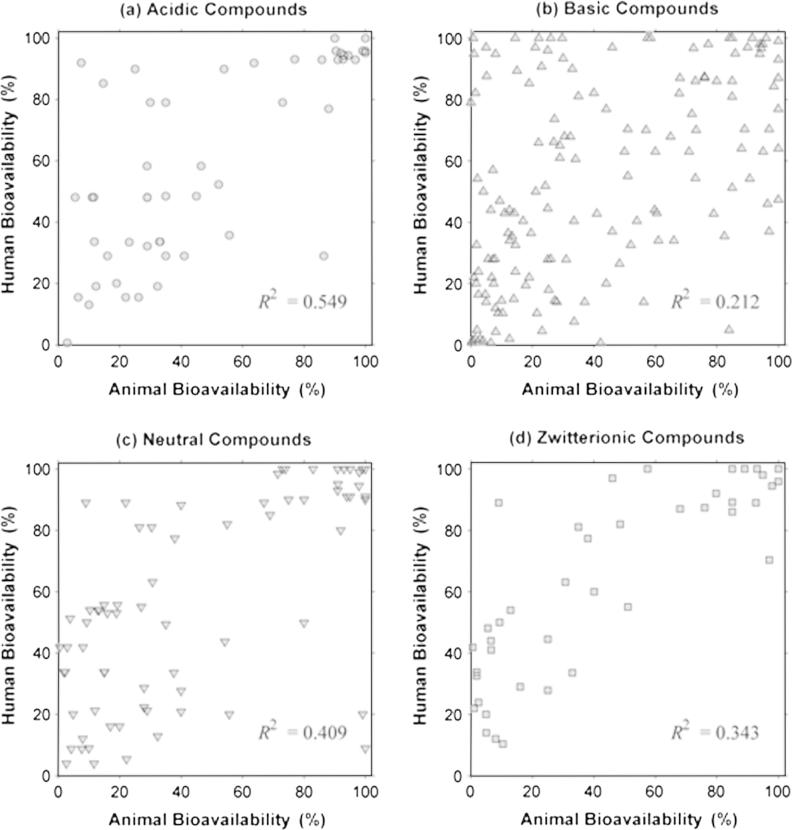
Plots for the linear regression analysis by separated by ion class (in percentages), the coefficient of determination (*R*^2^) for the linear regression are shown in each plot. (a) Mouse *F* vs. human *F*_;_ (b) Rat *F* vs. human *F*_;_ (c) Dog *F* vs. human *F* and (d) Non-human primates (NHP) *F* vs. human *F*.

**Fig. 5:**
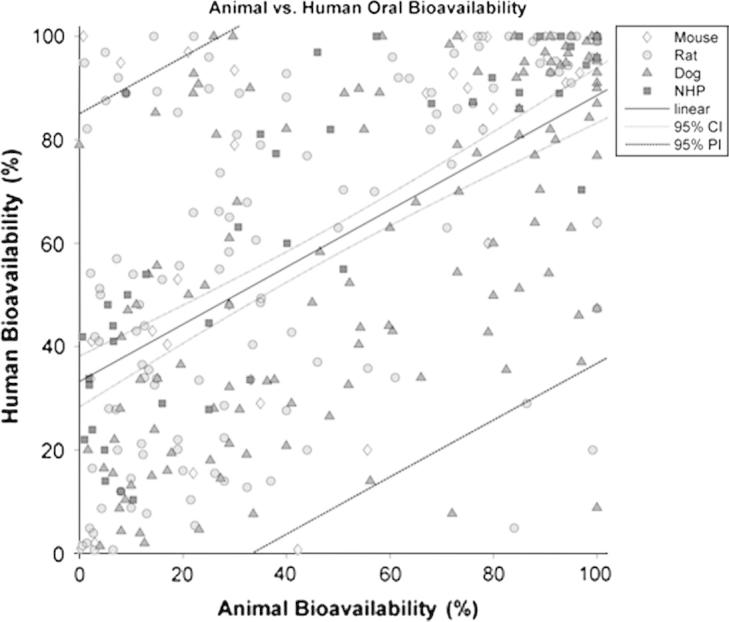
Plot of the linear regression analysis for the general dataset, animal vs. human oral bioavailability. Diamonds are for mouse, circles for rat, and triangles for dog and squares for non-human primates (NHP). Solid line (–), linear regression line; Pointed line (⋯), 95% confidence interval (CI) for mean response; Dashed line (- - -), 95% prediction interval (PI) for a future value.

**Fig. 6:**
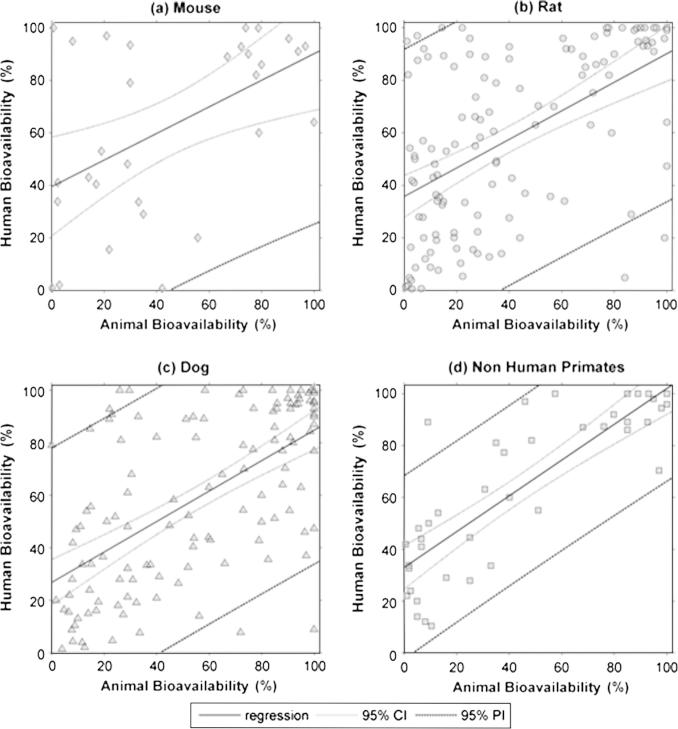
Plots for the linear regression analysis by classified by species (in percentages), the coefficient of determination (R^2^) for the linear regression are shown in each plot. (a) Mouse *F* vs. human *F*_;_ (b) Rat *F* vs. human *F*_;_ (c) Dog *F* vs. human *F* and (d) Non-human primates (NHP) *F* vs. human *F*_._ Solid line (–), linear regression line; Pointed line (⋯), 95% confidence interval (CI) for mean response; Dashed line (- - -), 95% prediction interval (PI) for a future value.

As shown in Table 4, linear regression analysis revealed a poor correlation for the overall animal and human oral bioavailability relationship (*R*^2^ = 0.342). Dog data, showed a minor improvement in the *R*^2^ value compared to the value from obtained from the whole dataset, whereas for the mouse and rat data, *R*^2^ values were lower than for the dog. In contrast, *R*^2^ value obtained for the NHP was higher than the value for the overall dataset and for every species in particular (Fig. 2). However, a prediction of *F_Human_* from *F_Animal,species_*, employing the linear regression model, resulted in wide prediction intervals (PI), as shown in Figs. 5 and 6. Afe calculations showed values below the unity for the general dataset and for every species in particular. Calculated values of the concordance correlation coefficient highlighted the lack of agreement between human and animal bioavailability for all species, suggesting a lack of precision in any quantitative prediction. In addition the median ratio between animal and human oral bioavailability (*R*_A/H_) showed similar results for the general dataset and almost all the species. The dog, however, showed a median value close to the unity (median *R*_A/H_ = 0.990), but with the highest interval of all the species (0.236 to 3.254 for the 5th and 95th percentile, respectively) (Fig. 3). A similar scenario occurs for the correlation analysis by ion class summarised in Table 2 and Fig. 4. Acidic drugs showed the highest *R*^2^ value (*R*^2^ = 0.549); followed by neutral and zwitterionic drugs, while the lowest *R*^2^ value was for basic compounds (*R*^2^ = 0.212) (see Tables 5 and 6).

Grouping compounds by formulation type (Solution or Solid) shows no advantage over the weighted combination of data with Solution and Solid showing similar *R*^2^ values (Solution *R*^2^ = 0.339, Solid *R*^2^ = 0.328) to the overall relationship, all of which indicate a poor correlation.

## 4 Discussion

The digitisation and careful re-analysis of the Grass and Sinko scatter plot raised a number of questions about the treatment of reported bioavailability data. Although it is clear that there are more data points (pair of human-animal bioavailability data) than compounds due to multiple comparisons, not all points could be readily identified in the associated database of Sitesema based on our re-analysis. No further detail on how the data were treated was given in the original review by Grass and Sinko and we could not resolve the disparities. It is plausible that multiple points are plotted to signify not just the mean values but also maximum and minimum reported values, where a large range of bioavailability had been observed. The combination of these factors (lack of clear description of the methodology and the apparent mismatch between the cited data and visualisation in the scatter plot) highlight anecdotal and occasional questions posed by those who believe animal data could be predictive of human bioavailability. These issues indicate that any comparisons between species should make an effort to clarify data extraction methodology and assumptions if the conclusions are to be used for defining drug development strategies with confidence.

Our methodology involved combining multiple studies by calculating a weighted mean which was less ambiguous when constructing scatter plots and correlations. However, it had the disadvantage that, where a large range of bioavailability values is reported, this is not captured in the correlations. Alternative strategies may involve separation of formulations, and a cursory analysis was undertaken utilising the new dataset and a reported formulation type (Solution or Solid). However, this is not an ideal scenario where the data in humans and animals are generated by different research groups using different materials but it is impractical to attempt to apply this formulation matching criteria due to the limitations it imposes on the dataset. Another consideration for correlations could be related to weighting each of the data points based on the numbers subjects and animals used for each combination.

The current dataset is, to the best of our knowledge, the largest dataset published for investigating the correlation between animal and human oral bioavailability. However, in addition to the complexity of the data analysis, the process of the new literature analysis illuminated further issues with performing a correlation of this magnitude on bioavailability data. For instance, we had to discard some of datapoints from previously published correlation studies due to more stringent inclusion criteria.

Clarity of the bioavailability studies in the literature varies widely. The information included in some publications provide the full details of equations employed, and they are transparent in describing methods of determination, formulations utilised and full details of subjects. However, some other reports provided minimal or in occasions no information in some aspects related to the study and data analysis. Tracing used reference in other literature back to the original study proved problematic in some cases, particularly where older papers were concerned. This led to exclusion of reports from current dataset when the original report was not available and not analysed by authors of the current report.

The regression analysis indicated that non-human primates are the most predictive amongst other species for human bioavailability (*R*^2^ of 0.7). Although the median relative bioavailability between animal species and human was unbiased for dog (median value of the ratio being close to the unity), the wide range indicated imprecision of the values as a predictive measure with confidence. The regression plot for dog (Fig. 2) highlights the above conclusion showing the large degree of scatter and the poor correlation coefficient. For rat and mouse there was no indication of any good correlation with the human data from any of the observed results. The results questioned the default assumption that bioavailability in rat or mouse can be a quantitative indication for human bioavailability.

The dataset for non-human primates was far more limited than for rat or dog, with only 41 data points available. This is not surprising considering the higher cost and more restricted ethical aspects associated with these studies. Further data could provide more confidence in relatively high correlations observed. However, range of predicted bioavailability from non-human primates compared to observed human values (Fig. 6) was wide and indicated the qualitative rather than quantitative value.

When the formulations were matched, there was still an apparent lack of correlation. In addition, when exploring the full dataset, due to the use of solutions as the main route of administration in animal studies (c.f. tablets in human), it might have been expected that a bias towards higher bioavailability values in animal should be seen. However, this was not the case, suggesting that the formulations did not have a significant impact on the correlations and bioavailability (note that all extended release formulations were excluded from the database). This confirms that the notion that other factors, such as metabolic differences between species, could play a more important role in defining disparities human vs. animal drug bioavailability. Accounting for such differences may improve understanding the differences and avoid over reliance on quantitative value of animal to human extrapolation of bioavailability.

## 5 Conclusion

An extended dataset to previously published reports was generated for animal vs. human bioavailability data with clear inclusion criteria. This highlighted that there are not strong and predictive linear correlations between overall and single species animal drug bioavailability and human values. Classification of high or low bioavailability could be achieved by setting certain cut-off points however quantitative models of oral drug bioavailability should be built for each species based on understanding the physiologic, metabolic and transporter related information affecting bioavailability.

## Figures and Tables

**Table 1:** Inclusion criteria for studies.

1. Oral and intravenous data should be established in the same group
2. Species should fall under category of Mouse, Rat, Dog or Non-Human Primate
3. AUC should be calculated to infinity or absorption phase should be complete
4. Original study data (no review articles) must be included when possible

**Table 2:** Points extracted from Grass and Sinko plot.

Species	Number of points expected	Number of points extracted
Rodent	40	61
Dog	43	76
Primates	11	15

**Table 3:** Interspecies oral bioavailability (*F*) for the selected compounds.

Compound Name	Ionic class	Mouse			Rat			Dog			NHP			Human		
		Mean F (%)	Range F	*n*	Mean F (%)	Mean F (%)	Range F	Mean F (%)	Range F	*n*	Mean F (%)	Range F	*n*	Mean F (%)	Range F	*n*
5-Fluorouracil	Neutral				28.0									28.6	26–33	3
Acarbose	Base							4.0						1.4		
Acebutolol	Base				61.0									34.0		
Acetylsalicylate (Aspirin)	Acid				35.0			45.0						48.5		
Acyclovir	Base				2.18			90.7						54.2		
Adefovir	Base				8.0									12.0		
Alprazolam	Acid				7.5									92.0		
Amitriptyline	Base							96.5						46.0		
Amlodipine	Base	100.0			100.0			88.0						64.0		
Amosulalol	Base				22.1			58.5			57.4			100.0		
Amoxicillin	Zwitterion							76.8						77.4		
Antipyrine	Neutral							73.0						100.0		
Azathioprine	Base										51.0			55.0		
Azithromycin	Base				46.0			97.0						37.0		
Cefixime	Acid							52.2						52.3		
Cefuroxime Axetil	Acid				23.1									33.5		
Chlorpheniramine	Base							9.4						47.0		
Clonazepam	Base							33.0						90.0		
Cyclosporine	Neutral							40.0						20.8		
Dapsone	Base							100.0						93.0		
Diazepam	Base							86.0						95.0		
Doxazosin	Base				50.0			60.0						63.0		
Erythomycin	Base				14.5	14–15	2	52.0			1.9			32.6	32–35	2
Estradiol Valerate	Neutral				4.3			7.8	0.3–9	2				8.7		
Estramustine phosphate	Neutral							54.3						43.7		
Ethambutol	Base										76.0			87.4	77.3–97.4	2
Ethimizol	Neutral				32.4									12.8		
Ethinylestradiol	Neutral				3.0			8.1	7.5–9	2	0.6			41.9	36–59	4
Ethosuximide	Neutral	74.0			83.0			91.0			93.0			100.0		
Ethylmorphine	Base				5.7			26.0						28.0		
Etoposide	Neutral				10.4	8.9–13.9	3	13.4			12.9			54.0	52–57.3	2
Felodipine	Neutral				20.0			17.0						16.0		
Fenfluramine	Base				15.0									89.3		
Fenoterol	Base				0.6	0.4–0.8	2							1.5		
Fexofenadine	Zwitterion	2.4			3.8	2.6–4.6	3				6.6			41.0		
Finasteride	Neutral							92.0						80.0		
Fleroxacin	Zwitterion	78.8			88.5			100.0						99.9	99–100	3
Fluconazole	Neutral	75.0			80.0			100.0						90.0		
Flumazenil	Neutral				28.0									22.3	15–27.8	2
Flunisolide	Neutral	55.7			99.2			55.0			4.9			20.0		
Flunitrazepam	Neutral				69.0									85.0		
Fluvastatin	Acid	35.0			86.4			41.0			16.0			29.0		
Foscarnet	Acid							10.0						13.1	9.1–17.1	2
Fosfomycin	Acid							29.0						32.2	28–37	2
Furosemide	Acid				28.9			46.5						58.3	43.4–71	8
Gabapentin	Zwitterion	79.0			79.0			80.0			40.1			60.0		
Ganciclovir	Neutral				10.0			100.0						8.8		
Gatifloxacin	Zwitterion				60.5			98.5						96.0		
Gitoxin	Neutral							91.1						95.0		
Glaziovine	Base							98.5						84.3		
Glyburide (Glibenclamide)	Acid							14.7						85.3	82–89	3
Griseofulvin	Neutral				3.9									51.2		
Guanfacine	Base										35.0			81.1		
Hydralazine	Neutral							37.7						33.6	31.3–35.4	2
Hydrochlorothiazide	Neutral										30.7			63.1	60.2–67.5	2
Ibuprofen	Acid				90.0						100.0			100.0		
Idazoxan	Base				12.6			66.0						34.0		
Ifosphamide/ Ifosfamide	Neutral				40.0									88.3		
Indapamide	Neutral							98.0						99.0		
Indomethacin	Acid				63.7									91.9	89–100	2
Isosorbide dinitrate	Neutral				40.0									27.6	19–48	6
Isosorbide-2-mononitrate	Neutral				100.0									100.0		
Isosorbide-5-mononitrate	Neutral							71.5						98.4	93–100	5
Isoxicam	Neutral							99.0						100.0		
Itraconazole	Neutral				27.1	16.6–34.9	2							55.0		
Ketanserin	Base				100.0			100.0						47.4	45–51	3
Ketorolac	Acid	90.4			99.1	91–100	2	100.0			100.0			95.9	80.5–100	2
Lansoprazole	Neutral				30.5			26.5						81.0		
Levodopa	Zwitterion							36.3						33.3		
Levofloxacin	Zwitterion				87.6									99.0		
Levonorgestrel	Neutral	67.0			9.0			22.0			9.0			89.0		
Lidocaine (Lignocaine)	Base				7.1			31.0			25.0	25–25	2	27.8	12.6–37	4
Linezolid	Neutral				95.0			95.0						100.0		
Lisuride	Base				28.0						5.0			14.0		
Lithium carbonate	Neutral										97.9			94.5		
Losartan	Acid				55.7									35.8		
Melagatran	Zwitterion				13.0			72.0						7.7		
Meloxicam	Neutral	94.0			95.0			100.0						91.0	89–97	2
Menogaril	Acid	33.2						11.8			33.0			33.6		
Mepindolol	Base				1.5			40.0						82.1		
Mercaptopurine	Neutral										8.0	4–12	2	12.0		
Metformin	Base				34.1									60.6		
Methadone	Base							0.0						79.0	79–79	2
Methylprednisolone	Neutral				35.1									49.4		
Metoclopramide	Base				71.9	49–91	2							75.3	58.1–84	4
Metolazone	Neutral							80.0						49.9		
Metoprolol	Base										25.0			44.5	41–50	5
Midazolam	Neutral	2.3			14.9	2.3–45	8	15.1			1.9	1.6–2.1	3	33.8	24–48	8
Morphine	Base				12.1	6.5–14.9	3	19.6	17.9–23	2				36.5	26–47.1	5
Moxifloxacin	Zwitterion	78.0			78.0			91.0			48.5	45–52	2	82.0		
Moxonidine	Base				5.1									87.6		
Nalbuphine	Base				2.5	0.9–4.7	3	4.8	3.5–5.6	2				16.4		
Naloxone	Base	0.3												0.9		
Naltrexone	Base	0.8			91.5									100.0		
Naproxen	Acid							100.0						100.0		
Naratriptan	Base				71.0			95.0						63.0		
Nefazodone	Base							14.0						15.0		
Nevirapine	Neutral				91.0									93.0		
Nicardipine	Base				21.5			8.9			10.4			10.4	10.2–10.5	2
Nifedipine	Neutral										9.3			50.0	43–63	7
Nimodipine	Neutral				22.3									5.4		
Nisoldipine	Neutral				2.7			11.7						3.9		
Nitrendipine	Neutral				12.0			29.0						21.2		
Nizatidine	Base				72.4			94.4						96.7	94–100	2
Nomifensine	Base							48.3						26.5		
Norfenfluramine	Base				19.0									85.3		
Nufenoxole (SC–27166)	Base				96.0			26.0			85.0			100.0		
Ofloxacin	Zwitterion				77.8									100.0		
Omeprazole	Neutral				19.3	6.4–40.8	2	15.0						55.6	40–70	3
Ondansetron	Base				7.2	6–8.6	2							57.0		
Oseltamivir acid	Acid	30.0			35.0			73.0						79.0		
Oxazepam	Zwitterion	72.3	56–88.5	2	40.0			22.1						92.8		
Phenobarbital	Acid	96.7						91.0						93.0	11–15.5	3
Phenoxymethylpenic–illin (Penicillin V)	Acid	29.0			11.6			11.0			5.5			48.1		
Phenytoin	Acid							36.0						78.5	69.9–90	3
Physostigmin	Base				2.0									4.9	3.2–8.2	2
Pindolol	Base	80.0			73.0			85.0			85.0			86.0		
Piroxicam	Zwitterion							100.0			89.0			100.0		
Pravastatin	Acid				12.3			32.3						19.1		
Prazosin	Base							30.5	23–38	2				68.0		
Prednisolone	Neutral							55.0						82.0		
Prednisone	Neutral										38.0			77.3	69–80	3
Primaquine	Base				25.0									96.0		
Procainamide	Base							85.0						80.9	75.3–83	2
Propoxyphene	Base							25.3						18.0		
Propranolol (±)	Base				19.0			6.8			<1			22.0		
Propranolol (−)	Base							7.9	5.7–10.5	2				28.0		
Propranolol (+)	Base							17.8	16.1–19.9	2				19.4		
Propylthiouracil	Acid							88.0						77.0		
Pyridostigmine	Base							33.6						7.6	14.3–7.6	2
Quinidine	Base				57.0			73.3						70.0		
Rabeprazole	Base							24.3						51.8		
Ranitidine	Base							73.0						54.4	52–60	2
Reboxetine	Base	21.0			5.0			90.0			46.0			97.0		
Recainam	Base				51.0			89.0			97.0			70.3	67–73	2
Remoxipride	Base	8.0			<1			94.0						94.9	93–96	2
Rifabutin	Base				44.0									20.0		
Rifampin	Zwitterion				89.0									94.8		
Risedronate	Acid				2.9									0.6		
Risperidone	Base				22.0									65.9		
Rosiglitazone	Zwitterion				100.0									99.0		
Rosuvastatin	Acid				19.0									20.1		
Salbutamol	Base							85.0						51.2	50–53	2
Salicylate	Acid				92.0			100.0						95.2		
Saquinavir	Base	42.2			6.5									0.7		
Selegiline	Base							8.1						4.3		
Sildenafil	Base	17.0			33.5			54.0						40.4	38–41	2
Sitafloxacin	Zwitterion				30.9			51.2			92.7			89.0		
Sitagliptin	Base				76.0			100.0			68.0			87.0		
Sotalol	Base							84.1						100.0		
Sparfloxacin	Zwitterion				61.6	58.3–63.3	2	84.4	77–91.9	2	79.7			92.0		
Sulfisoxazole	Zwitterion				77.0									100.0		
Sulpiride	Base				13.4			82.5						35.5		
Sumatriptan	Base				37.0			56.2	54–58	2				14.0		
Tacrolimus	Acid	22.0			26.2			6.5						15.5	15–17.8	2
Talinolol	Base				29.0	17.1–52.1	3							65.0	55–68.9	2
Tamsulosin	Base				14.4			29.7						100.0		
Terazosin	Base				67.8									82.0		
Terodiline	Base							23.0						90.8	90–92	2
Tetrabenazine	Base				84.0									4.9		
Theophylline	Base				77.2	63.8–97.2	3	94.0	91–100	2	94.9			98.0	94–100	5
Tiagabine	Acid				25.0			54.0						89.9		
Timolol	Base							29.0						61.0		
Tinidazole	Base							100.0						99.0		
Tolterodine	Base	14.2			11.0			60.5						43.0		
Torsemide	Acid				92.7	87.5–95.6	2	77.0						93.1	89–96	2
Tramadol	Base				32.4			65.0						67.9		
Trazodone	Base				27.2									73.6	63–77	2
TRH Tartrate	Base				1.5			12.6						2.0		
Triazolam	Neutral	19.0			16.0									53.0		
Trovafloxacin	Zwitterion				68.0			58.0			85.0			89.1	87.6–91	2
Valproic Acid	Acid				94.4			93.0						94.4	92.8–96	2
Vardenafil	Base				10.0			27.3						14.5		
Venlafaxine	Base				12.6			59.8			6.5			44.0		
Verapamil (±)	Base							14.5	13.8–15.2	2	2.5			23.9	18–38	3
Verapamil (−)	Base				7.4			1.7	1.5–1.9	2				20.0		
Verapamil (+)	Base				4.1			21.1	20.6–21.6	2				50.0		
Warfarin	Acid							85.8						93.0		
Xamoterol	Base							23.2	8–34	2				4.6	4.5–4.8	3
Zalcitabine	Base	30.0												93.4	86–100	3
Zanamivir	Base	3.0												2.0		
Zolmitriptan	Base				41.0			79.0						42.8	39–49	2
Zolpidem	Base				27.0									66.2	65.8–66.6	2
Zopiclone	Base				44.0			100.0						76.9	76.7–77	2

*F*, oral bioavailability; Range, *F*, range for the mean bioavailability values for the studies; *n*, number of studies for the calculation of the weighted mean. References for the bioavailability studies can be found in the supplementary material for the online version of this article.

**Table 4:** Linear regression analysis, afe and animal/human oral bioavailability ratio.

Species	Number of points	Slope (*β*)	Intercept (*α*)	*R*^2^	ccc	afe	Median *R*_A/H_	5% lower percentile for *R*_A/H_	95% upper Percentile for *R*_A/H_
All	318	0.553⁎	33.114	0.342	0.548	0.647	0.866	0.082	2.771
Mouse	30	0.507⁎⁎	39.478	0.253	0.444	0.593	0.784	0.058	2.784
Rat	122	0.544⁎	35.759	0.287	0.470	0.583	0.723	0.075	2.777
Dog	125	0.580⁎	26.433	0.374	0.605	0.845	0.990	0.236	3.254
NHP	41	0.691⁎	32.942	0.694	0.698	0.417	0.592	0.051	1.042

Regression equation, *F_Human_* = *β* * *F_Animal,species_* + *α*; *R*^2^, coefficient of determination; ccc, concordance correlation coefficient; afe, average fold error; *R*_A/H_, animal and human oral bioavailability ratio.

⁎ *p* value vs. constant model < 0.001.

⁎⁎ *p* value vs. constant model < 0.005.

**Table 5:** Linear regression analysis by ionic class.

Species	Number of points	Slope (*β*)	Intercept (*α*)	*R*^2^
Acid	53	0.686⁎	26.295	0.549
Base	152	0.440⁎	35.891	0.212
Neutral	73	0.596⁎	30.897	0.409
Zwitterion	39	0.524⁎	44.592	0.343

Regression equation, *F_Human_* = *β* * *F_Animal,ionclass_* + *α*; *R*^2^, coefficient of determination.

⁎ *p* value vs. constant model < 0.001.

**Table 6:** Linear regression analysis by formulation type.

Species	Number of points	Slope (*β*)	Intercept (*α*)	*R*^2^
Solution	57	0.157	32.128	0.339
Solid (Capsule/Tablet/Solid)	30	0.524	34.153	0.328

Regression equation, *F_Human_* = *β* * *F_Animal,formulation_* + *α*; *R*^2^, coefficient of determination.

## Appendix A Supplementary material

Supplementary data 1 The detailed dataset for the compounds listed in Table 3, including some physico-chemical properties, formulation details, and references for the oral bioavailability values, in both human and preclinical species, can be found as an electronic supplementary material in the online version of this article.
